# Supplementary material for: Development of swarm behavior in artificial learning agents that adapt to different foraging environments
Source: PLoS One. 2020 Dec 18;15(12):e0243628. doi: 10.1371/journal.pone.0243628 (PMC7748156; doi:10.1371/journal.pone.0243628)
Supplement: S1 Appendix — In this appendix, we provide additional information about the dynamics presented in Sec. 3 of the main text. In particular, we analyze why there is transition at dF = 6 from the regime where agents form cohesive swarms to the one in which they form aligned swarms to reach the food source. Furthermore, we extend the analysis of alignment and cohesion presented in Sec. 3.2 of the main text. (PDF) [file pone.0243628.s001.pdf]

S1 Appendix

Additional analysis

Transition from cohesive to aligned dynamics

First, we analyze in detail why there is a transition at $d_F \simeq 6$ (see Figs. 7 and 14 of the main text) from the regime where cohesive swarms emerge to the regime where aligned swarms emerge as a result of the learning processes. We attribute this phenomenon to the fact that the agents are initialized in a region of size $2V_R$ (12 in our case), which means that a food source placed at $d_F = 6$ is exactly at the edge of this region. Consider the case where the food is placed inside the initialization region: in this case, it is most likely that agents will find the food—which is the condition for being rewarded— while they are surrounded by many neighbours. Consequently, behaviors that entail approaching or staying with other agents are more likely to lead to rewards —effectively, agents learn to 'join the crowd'. However, if the food is placed outside the initial region, agents need to leave regions where the density of agents is high at the beginning of the trial, but they also need to stabilize their orientations, which is best achieved by aligning with one's neighbors. We have tested this hypothesis by changing the initial region. Figs. 1 and 2 of this appendix show analogous data to Figs. 7 and 14 of the main text, but with agents initialized in the first V_R positions of the world (half of the previous region). We observe that the transition in behavior happens at $d_F = 3$ in this case, which is the edge of the initial region.

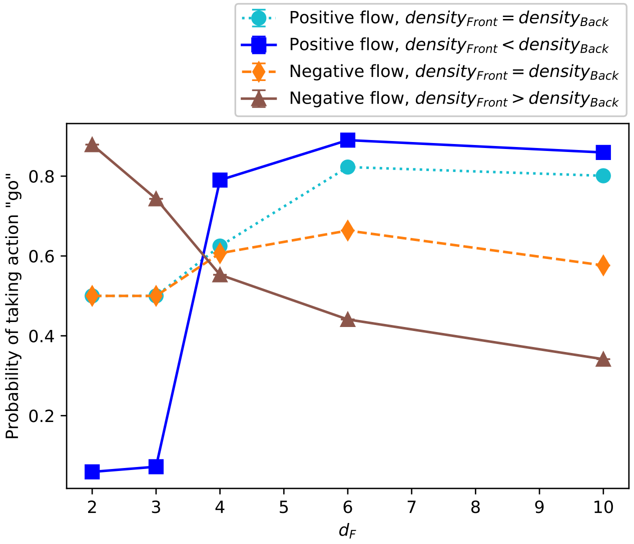

Fig 1. Final probability of taking the action "go" depending on the learning task (increasing distance to food source d_F) for four significant percepts (see legend). Average is taken over one ensemble consisting of 60 agents.

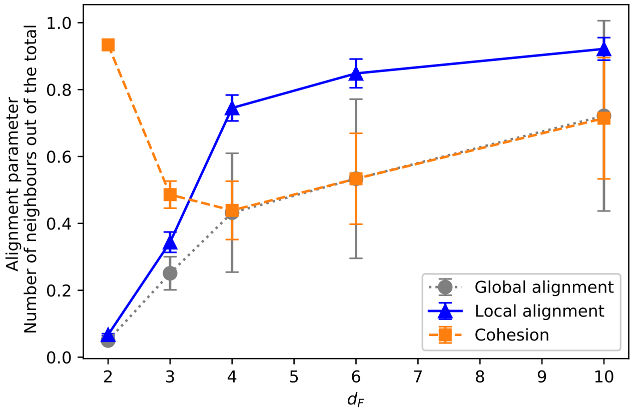

Fig 2. Average number of neighbors (as a fraction of the total ensemble size), global and local alignment parameter as a function of the distance d_F to the point where food is placed during training. Each point is the average of the corresponding parameter over all interaction rounds (50) of one trial, and then over 100 trials. One trained ensemble of 60 agents is considered for each value of d_F.

Details on analysis of alignment

In this section, we elaborate more on the splitting of the swarm that we observe in some of the trials for training with $d_F = 21$. In order to study this, we perform a simulation of 100 trials with ensembles of agents that are already trained with $d_F = 21$. Figure 3 (a) shows that, in some of these trials, almost all agents form one big swarm[1] ($\phi \simeq 0.85$) that goes in one direction, with few agents moving away from the swarm (grey histogram), whereas in other trials they form two swarms ($\phi \simeq 0.55$), roughly of similar size, that travel in opposite directions (pink histogram). Locally, agents are strongly aligned, as can be seen in Fig. 14 of the main text, where average local alignment parameter reaches 0.9 for $d_F = 21$. For $d_F = 6$, the swarm behavior is similar to the one observed for $d_F = 21$ (see Fig. 3 (b)), but the local alignment is not so strong, so there are more agents that go out of the swarm. For swarms trained with $d_F = 4$ (Fig. 3 (c)), we observe that there is no splitting and agents do not move beyond the initial region.

Details on analysis of cohesion

In this section, we provide an additional plot (Fig. 4) of the evolution of the local alignment parameter through the learning process for $d_F = 4, 21$. We observe that the increase of the local alignment parameter from trial 100 to trial 200 is the reason why the average number of neighbors decays at these same trials (see Fig. 11 of the main text). At these trials, agents have not yet learned to form swarms, but some of them have learned to go straight and started to learn to align with their neighbors. Thus, these agents are already able to go away from the initial region where the rest of agents are still doing a random walk. Consequently, these agents in particular have fewer neighbors, which reduces the overall average number of neighbors. For higher values of the local alignment parameter, as seen from trial 200 onwards, agents start to form strongly aligned swarms, which increases cohesion and consequently the number of neighbours M.

[1] We take the threshold for 'a single swarm' to be that 75% of agents move in the same direction.

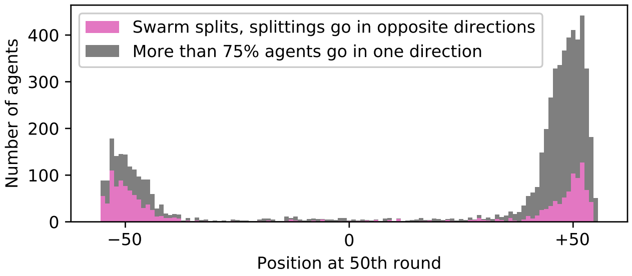

(a) Agents trained with $d_F = 21$

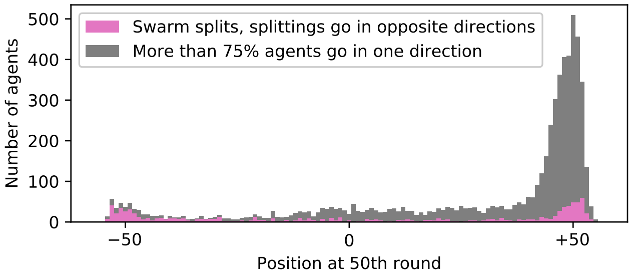

(b) Agents trained with $d_F = 6$

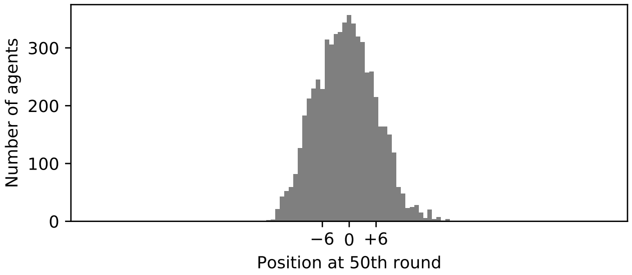

(c) Agents trained with $d_F = 4$

Fig 3. Stacked bar graph showing the number of agents that are located at a given position at the end of one trial (at the 50th interaction round). The graph is centered in C, which is the middle of the initial region (value 0 in the horizontal axis). Each data set (for each trial) is processed such that the majority of agents travel to the positive side of the horizontal axis. 100 trials of (already trained) ensembles of 60 agents are considered (one ensemble per trial). (a) Out of the 100 ensembles, 72 travel as one big swarm (grey) and 28 split into two subswarms that go in opposite directions (pink). In order to show that these are complementary subsets of the data, grey bars are *stacked* on top of pink bars. (b) Out of the 100 ensembles, 83 travel as one big swarm (grey) and 17 split in two subswarms that go in opposite directions (pink). (c) All ensembles are strongly cohesive and do not split. Agents do not travel beyond the initial region (marked in the horizontal axis).

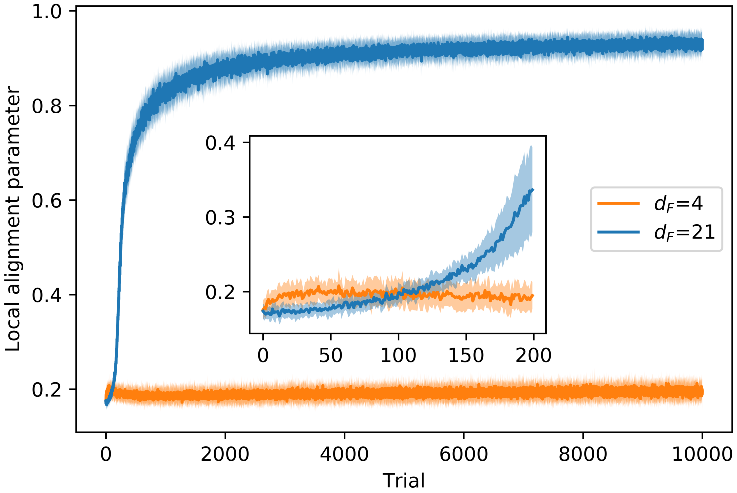

Fig 4. Evolution of the local alignment parameter through the learning processes with $d_F = 4, 21$. Each point is the average of the corresponding parameter over all interaction rounds (50) of one trial. 20 independently trained ensembles of 60 agents each are considered for the average.
