# Supplementary material for: Development of swarm behavior in artificial learning agents that adapt to different foraging environments
Source: PLoS One. 2020 Dec 18;15(12):e0243628. doi: 10.1371/journal.pone.0243628 (PMC7748156; doi:10.1371/journal.pone.0243628)
Supplement: S2 Appendix — We describe the statistical methods applied to obtain the results of Sec. 4. First, we explain how to optimize the likelihood function of each of the four models we consider, with a focus on the CCRW model. In addition, we describe the goodness-of-fit tests that we perform, that is, a log-likelihood ratio for the BW, CRW and PL models and a test based on uniform pseudo-residuals for the CCRW model. (PDF) [file pone.0243628.s002.pdf]

S2 Appendix

Statistical methods

The statistical methods we have applied in the main text are explained and detailed in this section. The statistical analysis consists of three steps: (i) the MLE estimation of the model parameters, for all models; (ii) the best model selection by means of the AIC and (iii) the absolute fit tests for the best model.

Likelihood functions and MLE

The expression of the likelihood function,

$$\mathcal{L}(\theta|\ell_{i=1..S}) = \prod_{i=1}^{S} p\left(\ell_i, \theta\right), \tag{1}$$

can lead to computational underflows when the sample size is large[1], so the usual procedure is to maximize its logarithm instead (which leads to the same result since this function is monotonic). For i.i.d. variables, the log-likelihood function has the simple expression,

$$\ln \mathcal{L}(\theta|\ell_{i=1..S}) = \sum_{i=1}^{S} \ln p\left(\ell_i, \theta\right), \tag{2}$$

where S is the sample size and $p(\ell_i, \theta)$ is the PDF of the given model —that depends on the model parameters θ— evaluated at the data point ℓ_i.

The first three models have i.i.d. variables, so the computation of their log-likelihood functions is straightforward once the PDFs of each model are defined (see main text for details). However, the log-likelihood function of the CCRW model cannot be expressed as a sum of the logarithms of the PDFs evaluated at each data point. From eq. (1), the expression in the case of the CCRW can be written as,

$$\mathcal{L}(\delta, \lambda_I, \lambda_E, \gamma_{II}, \gamma_{EE}|\ell_{i=1..S}) = \delta_M P(\ell_1) \prod_{i=2}^{S} \Gamma P(\ell_i) \mathbf{1}, \tag{3}$$

where,

$$\delta_M = \begin{pmatrix} \delta & 1-\delta \end{pmatrix}, \tag{4}$$

$$P(\ell) = \begin{pmatrix} p_I(\ell) & 0 \\ 0 & p_E(\ell) \end{pmatrix}, \tag{5}$$

$$\Gamma = \begin{pmatrix} \gamma_{II} & 1-\gamma_{II} \\ 1-\gamma_{EE} & \gamma_{EE} \end{pmatrix}, \tag{6}$$

$$\mathbf{1} = \begin{pmatrix} 1 \\ 1 \end{pmatrix}, \tag{7}$$

[1] Large product of terms below one gives values close to zero.

and the expressions for $p_I(\ell)$ and $p_E(\ell)$ are given in eqs. (10) and (11) of the main text (note that they depend on λ_I and λ_E, respectively). Since the variables are not independent in this case, the log-likelihood function cannot be directly obtained with expression (2). In addition, function (3) cannot be directly computed due to underflow errors. To avoid this, we apply the techniques explained in chapter 3 of [1] (specifically, the algorithm for the computation of the log-likelihood function given in appendix A.1.3 of [1]).

Once the log-likelihood functions are computed, the maximization (minimization of the negative log-likelihood function) with respect to the model parameters is performed using the Python function scipy.optimize.minimize. The MLE parameters obtained for each model are given in the main text. The MLE of the minimum step length can be directly considered to be the observed one [2] (in our case it is $\ell = 1$ since agents move one position per interaction round).

Goodness-of-fit tests

In this work, we have performed two types of goodness-of-fit (GOF) tests; one for the models with i.i.d. variables (BW, CRW and PL) and a different one to account for the temporal autocorrelation of the CCRW model. For the BW, CRW and PL models, we apply a likelihood ratio test to compare the likelihood of the observed frequencies to the likelihood of the theoretical distribution that corresponds to the given model. More specifically, we compute the log-ratio [3],

$$\mathcal{R} = \sum_{i=1}^{S} [\ln f_{obs}(\ell_i) - \ln f_{th}(\ell_i)], \tag{8}$$

where S is the sample size and f_{obs}, f_{th} are the observed and theoretical frequencies of the ith step length, respectively. Note that the theoretical frequency is just the probability (see main text for the expressions of each model's PDF) of the ith step length times the sample size S.

Normally, likelihood ratios like $\mathcal{R}$ above are used to compare two competing theoretical models, in which case a large absolute value of $\mathcal{R}$ indicates that one model is clearly better than the other. In order to assess how much better it is, one asks how likely it is that a given absolute value of $\mathcal{R}$ could have arisen purely from chance fluctuations, if in fact both models were equally good. This is quantified by the *p-value* (App. C, eq. (C.6) of ref. [3]). When one compares two theoretical models, finds a large $|\mathcal{R}|$ and its corresponding p-value is small, this indicates that the value $\mathcal{R}$ is unlikely to be a chance fluctuation, and that one can therefore exclude one model with high confidence.

In our case, however, a good fit between the theoretical model and the observed frequencies manifests as small $|\mathcal{R}|$ and correspondingly large p. Small p-values, on the other hand, indicate that it is unlikely that the data were generated by the proposed model. One can therefore interpret $1 - p$ as the probability with which we can rule out the proposed theoretical model. The p-values obtained in our analysis are given in Fig. 1.

In the case of the CCRW model, one cannot directly perform a GOF test on the raw data points due to the autocorrelation present in the HMM model.

We circumvent this problem using *pseudo-residuals*, as described in [1]. Given a continuous random variable X and a function $F(X)$ defined by the cumulative distribution function (CDF),

$$F(X = x) := Pr(X \leq x), \tag{9}$$

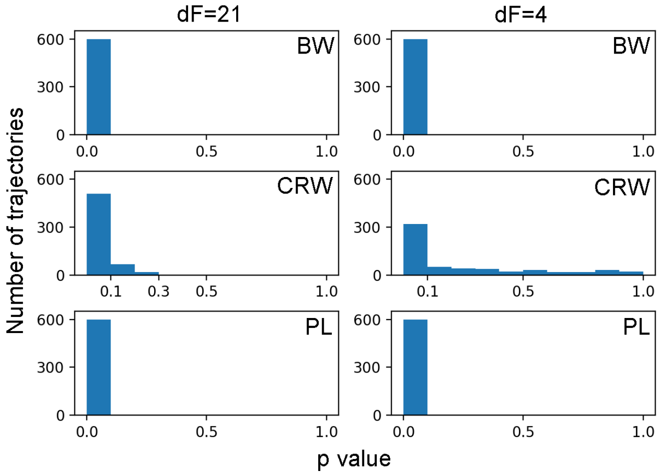

Fig 1. Histograms of the p-values obtained for the BW, CRW and PL models in the $d_F = 21$ and $d_F = 4$ cases. In our goodness-of-fit test, p-values close to zero rule out the proposed theoretical model, while values close to 1 represent compatibility with the model.

the pseudo-residual u is obtained by sampling a value x of X, then taking the corresponding value of the function F. If X is sampled from some probability distribution P_{exp} and we take F_{exp} to be the CDF of that same distribution,

$$F_{exp}(X = x) = \int^{x} P_{exp}(X = x')dx', \tag{10}$$

then one can show that the resulting probability distribution over the pseudo-residuals is in fact uniform $U(0, 1)$ [1]. If, on the other hand, we take F_{theo} to be the CDF derived from some proposed theoretical distribution P_{theo}, then the pseudo-residuals will in general not be uniformly distributed. By testing whether the pseudo-residuals with respect to a given theoretical model are uniformly distributed, one can therefore test whether the model is a good fit for the data.

In order to accommodate discrete variables, one introduces so-called mid-pseudo-residuals,

$$u^m = (u + u^-)/2, \tag{11}$$

where u is obtained by sampling a value x of X and taking the corresponding $F(X = x)$, as above, while $u^- = F(X = x^-)$ is the value of F at the greatest possible realization that is strictly less than the sampled x.

Our data consists of a time-series of step lengths ℓ_t, each of which gives rise to one mid-pseudo-residual u_t^m. Therefore, the first step length is denoted ℓ_1 and the last one ℓ_S, since S is the sample size. In order to be consistent with the notation used in the main text for step lengths, we use in the following the upper case L to denote the random variable and the lower case ℓ to denote one realization of it.

Crucially, the probability distribution over step lengths at each time-step is different, since it is correlated with the lengths of preceding steps:

$$u_t^- = \Pr(L_t < \ell_t | L^{(-t)} = \ell^{(-t)}), \tag{12}$$

$$u_t = \Pr(L_t \leq \ell_t | L^{(-t)} = \ell^{(-t)}), \tag{13}$$

where the expression for the conditional probability ([1], (Chapter 5)) is in our case,

$$\Pr(L_t \leq \ell | L^{(-t)} = \ell^{(-t)}) = \frac{\delta_M P(\ell_1) B_2 ... B_{t-1} \Gamma Q(\ell) B_{t+1} ... B_T \mathbf{1}}{\delta_M P(\ell_1) B_2 ... B_{t-1} \Gamma B_{t+1} ... B_T \mathbf{1}}, \tag{14}$$

where δ_M, $P(\ell)$, Γ and $\mathbf{1}$ are defined in eqs. (4), (5), (6) and (7) respectively and,

$$B_t = \Gamma P(\ell_t), \tag{15}$$

$$Q(\ell) = \begin{pmatrix} q_I(\ell) & 0 \\ 0 & q_E(\ell) \end{pmatrix}, \tag{16}$$

where $p_I(\ell)$ and $p_E(\ell)$ are the PDFs defined in the main text and $q_I(\ell)$ and $q_E(\ell)$ are their corresponding CDFs, respectively. Note that, in this expression, the parameters of the model are fixed (MLE parameters). Again in this case, a rescaling is needed in order to avoid underflows in the computation (see algorithm in App. A.2.9 of ref. [1]).

In summary, we first compute the mid-pseudo-residual for each data point and then we perform a GOF test on them. Since the probability distribution of the mid-pseudo-residuals approaches that of a continuous variable, one can apply a Kolmogorov-Smirnov (KS) test to check for uniformity. The KS statistic computes the distance (D_{KS}) between the CDF of the empirical data (in this case, the values u_t^m) and the CDF of the reference distribution (in this case, $U(0,1)$). Therefore, a value $D_{KS} = 0$ means that the data is distributed exactly as the reference distribution. The maximum KS distance is $D_{KS} = 1$. One obtains one value of D_{KS} for each individual trajectory (we perform the analysis on 600 trajectories for each type of swarm dynamics). The average value of the KS distance that we have obtained is $D_{KS} = 0.189 \pm 0.046$ for the trajectories of agents trained with $d_F = 21$ and $D_{KS} = 0.134 \pm 0.016$ for the ones of agents trained with $d_F = 4$. All the values of D_{KS} are displayed in a histogram form in Fig. 2.

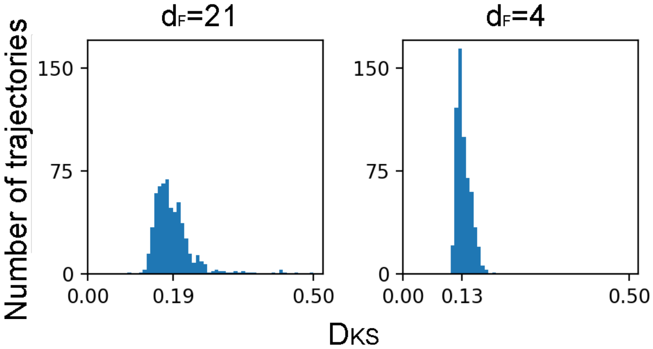

Fig 2. Histograms of the D_{KS} distances obtained in the GOF test of the CCRW model, for (left) $d_F = 21$ and (right) $d_F = 4$.

Additional tables and figures

Examples of the results of the statistical analysis for one trajectory are given in Tables 1, 2, and 3. The trajectories considered correspond to the ones displayed in Fig. 20 (b) of the main text, and Figs. 3 and 4 of this section, respectively. In addition, figures 3 and 4 provide the survival distributions of the trajectories that have the best goodness-of-fit parameter for the CCRW and the PL models, respectively.

Table 1. Results of the statistical analysis of the trajectory from Fig. 20 (b). This individual was chosen for achieving the closest fit to the BW and CRW models of all agents trained with $d_F = 4$.

Model	k	AIC	Δ_i	w_i	p-value
BW	2	130534.03	0	0.87	0.0018
CRW	4	130538.03	4	0.12	0.96
PL	2	144670.67	14136.64	0	< 0.01
CCRW	6	130542.03	8	0.01	$D_{KS} = 0.18$

Table 2. Results of the statistical analysis of the trajectory from Fig. 3. This individual was chosen for achieving the closest fit to the CCRW model of all agents trained with $d_F = 21$.

Model	k	AIC	Δ_i	w_i	p-value
BW	2	87207.06	2104.71	0	< 0.01
CRW	4	85676.13	573.78	0	< 0.01
PL	2	94815.86	9713.51	0	< 0.01
CCRW	6	85102.35	0	1	$D_{KS} = 0.094$

Table 3. Results of the statistical analysis of the trajectory from Fig. 4. This individual was chosen for achieving the closest fit to the PL model of all agents trained with $d_F = 21$.

Model	k	AIC	Δ_i	w_i	p-value
BW	2	85615.24	28236.12	0	< 0.01
CRW	4	58473.79	1094.67	0	< 0.01
PL	2	57379.12	0	1	< 0.01
CCRW	6	58471.36	1092.24	0	$D_{KS} = 0.29$

References

1. Zucchini W, MacDonald IL. Hidden Markov models for time series: an introduction using R. vol. 110. Monographs on Statistics and Applied Probability, CRC Press; 2009. Available from: `https://doi.org/10.1201/9781420010893`.

2. Edwards AM, Freeman MP, Breed GA, Jonsen ID. Incorrect likelihood methods were used to infer scaling laws of marine predator search behaviour. PloS one. 2012;7(10):e45174. doi:10.1371/journal.pone.0045174.

3. Clauset A, Shalizi CR, Newman MEJ. Power-law distributions in empirical data. SIAM review. 2009;51(4):661–703. doi:10.1137/070710111.

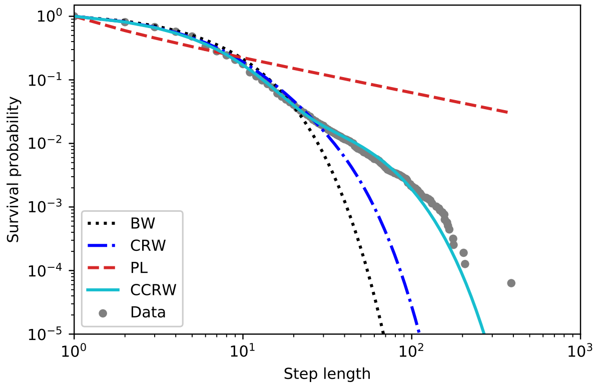

Fig 3. Survival probability (cumulative percentage of step lengths larger than the corresponding value in the horizontal axis) as a function of the step length. Trajectory of one agent trained with $d_F = 21$, which has an Akaike value of 1 for the CCRW model. This individual was chosen for achieving the closest fit to the CCRW model of all agents trained with $d_F = 21$. The survival distributions of the four candidate models are also plotted. The distributions for each model are obtained considering the MLE parameters.

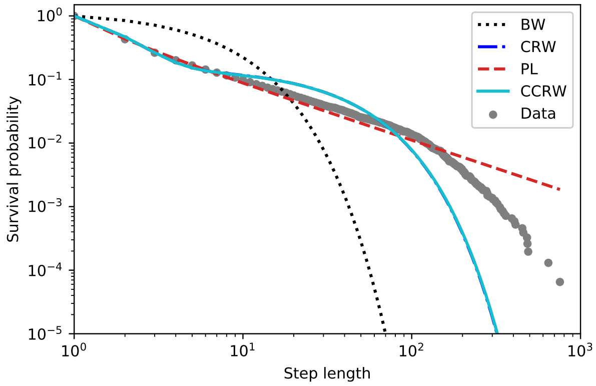

Fig 4. Survival probability (cumulative percentage of step lengths larger than the corresponding value in the horizontal axis) as a function of the step length. Trajectory of one agent trained with $d_F = 21$, which has an Akaike value of 1 for the PL model. This individual was chosen for achieving the closest fit to the PL model of all agents trained with $d_F = 21$. The survival distributions of the four candidate models are also plotted. The distributions for each model are obtained considering the MLE parameters.
